# The case report of capillary leakage syndrome secondary to malignant hypertension

**DOI:** 10.1097/MD.0000000000011913

**Published:** 2018-08-24

**Authors:** Xuejiao Liu, Guoqin Wang, Lijun Sun, Hongrui Dong, Yipu Chen, Hong Cheng

**Affiliations:** Department of Nephrology, Beijing Anzhen Hospital, Capital Medical University, Beijing, China.

**Keywords:** acute kidney injury, capillary leakage syndrome, edema, hypoalbuminemia, malignant hypertension

## Abstract

**Introduction:**

Capillary leak syndrome (CLS) is characterized by hypoproteinemia, diffused pitting edema, noncardiogenic pulmonary edema, and hypotension. By far, there are no related reports of CLS secondary to malignant hypertension (MHT). A 33-year-old male was admitted to our hospital with the diagnosis of CLS on the background of MHT.

**Patient concerns:**

A 33-year-old male was admitted with a 6-day history of worsening dyspnea, chest distress, and diffused pitting edema accompanied by very high blood pressure (200/145 mm Hg).

**Diagnoses:**

The tests and examinations showed hypoalbuminemia (26.7 g/L), pulmonary edema, and normal heart function. However, the expected massive proteinuria was absent (1.5 g/24 h). After diuretic and other antihypertensive therapy, the blood pressure reduced gradually; meanwhile, the symptoms of dyspnea and chest distress were improved quickly, and edema in his legs was also reduced. It is surprising that there was no change of pulmonary edema signs on imaging scan, and hypoalbuminemia remained with only mild proteinuria. Thus, our provisional diagnosis of this patient was CLS secondary to MHT.

**Interventions and outcomes:**

We administered intravenous immunoglobulin, sulodexide, and renin-angiotensin system inhibitor to the patient for repairing vascular endothelium and improving the function of vascular endothelium. Before discharge, the patient's edema disappeared and the chest X-ray turned to normal. The level of serum albumin also increased to 35.1 g/L along with the overall improvement. Finally, the renal biopsy revealed malignant hypertensive glomerulosclerosis. All these clinical manifestations were consistent with CLS caused by MHT.

**Lessons:**

Up to now, there has been no case report of CLS caused by MHT. We should pay more attention to CLS induced by MHT, try to diagnose it as soon as possible, and give prompt treatment to CLS and primary disease.

## 1 Introduction

Malignant hypertension (MHT) is defined as very high blood pressure (BP; diastolic BP ≥ 130 mm Hg), accompanied by bilateral hemorrhage or exudate, papilledema on fundoscopy. MHT is frequently complicated with renal dysfunction. The main manifestations are proteinuria with/without microhematuria, and acute renal insufficiency.^[1]^ Capillary leak syndrome (CLS) is characterized by hypoproteinemia, diffused pitting edema, exudative serous cavity effusion, noncardiogenic pulmonary edema, hypotension, and, in some cases, even hypovolemic shock. Massive proteinuria and nephrotic syndrome are rarely seen in CLS. Sepsis is the most commonly associated disease with CLS.^[2]^ Up to now, there has been no related report of capillary leakage syndrome secondary to MHT.

## 2 Case presentation

The study was approved by the Ethics Review Committee of Beijing Anzhen Hospital, Capital Medical University, and implemented in accordance with the Declaration of Helsinki. The patient has given consent to publish the case.

A 33-year-old male was admitted with a 6-day history of worsening dyspnea, chest distress, and diffused pitting edema accompanied by very high BP (200/145 mm Hg). The patient had gained 10 kg weight from 82 to 92 kg in 6 days. He had not visited any primary care physicians for many years, and had no medical history, even of hypertension. On examination, he was found to be severely hypertensive (as high as 200/145 mm Hg), hypoxemia (the finger oxygen saturation was 93% on room air), and with normal temperature. He was in semidecubitus position, with dependent-pitting edema from foot to abdomen, as well with palpebral edema.

**Figure 1:**
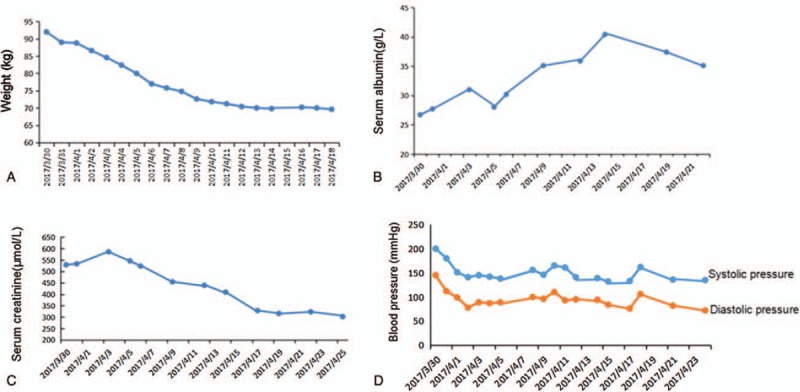
Trend of clinical values of this patient. In hospital, the change of patient's (A) weight, (B) serum albumin, (C) creatinine, and (D) blood pressure.

**Figure 2:**
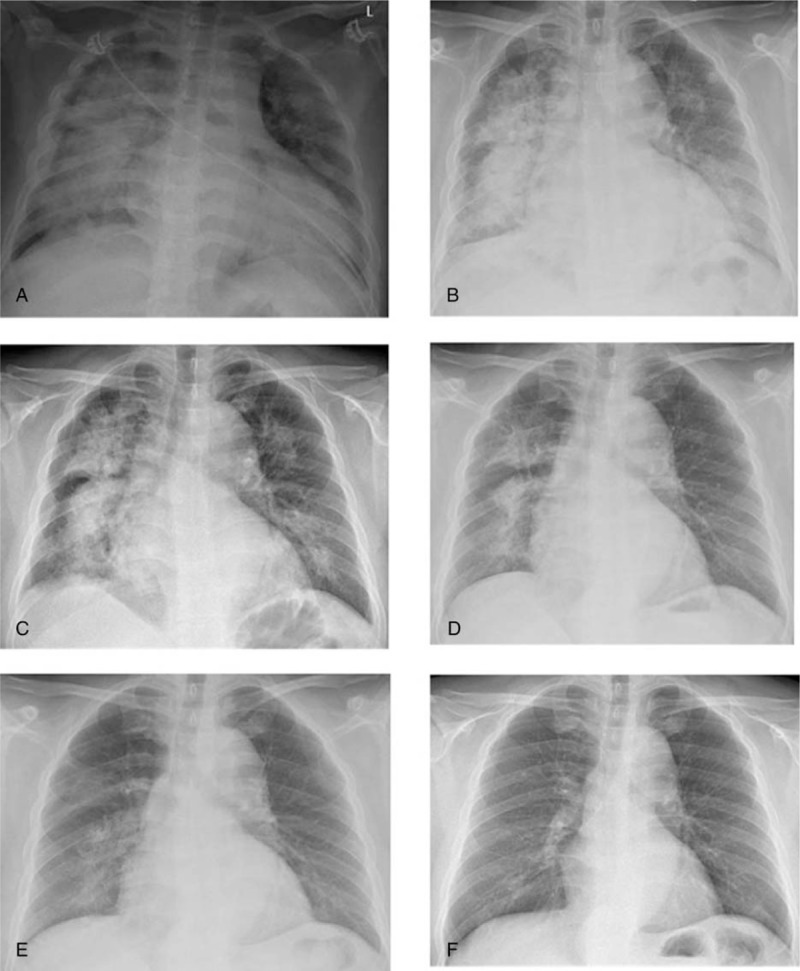
The chest X-ray manifestations of this patient. Since the first visit, the change of the patient's chest X-ray manifestations on the (A) 1st day, (B) 3rd day, (C) 1st week, (D) 2nd week, (E) 4th week, and (F) 8th week.

The tests (Fig. 1) showed elevated serum creatinine (529 μmol/L) and urea nitrogen (38 mmol/L), hypoalbuminemia (26.7 g/L), but mild proteinuria (1.5 g/24 h). The white cell count was normal and all bacteriologic samples were sterile. Fundus examination found grade III hypertensive retinopathy. On chest X-ray, imaging changes were compatible with pulmonary edema (Fig. 2A) and pulmonary function test revealed impaired diffusion function. Trans-thoracic echocardiogram (TTE) illustrated a normal left ventricular ejection fraction (LVEF) of 67%, and there were neither acute apical ballooning of the left ventricle nor signs of segmental abnormality. Serial electrocardiograms and cardiac troponin samples excluded myocardial infarction.

Thus, he was commenced on diuretic therapy and other anti-hypertensive therapy (beta-blocker and calcium channel blocker) immediately. The patient's symptoms improved quickly in 3 days, as the drop of BP, the symptom of dyspnea, and chest distress were improved quickly. The edema in his legs was also reduced, and he lost 3 kg body weight (Fig. 1A).

However, it is surprising that the chest X-ray showed no improvement (Fig. 2B) with pulmonary edema, and the hypoalbuminemia could not be explained by mild proteinuria. Thereby, in addition to diuretic therapy, one dose of intravenous immunoglobulin (IVIg; 5 g) was administrated for attempt; meanwhile sulodexide was also prescribed to for repairing vascular endothelium and improving the function of vascular endothelium. After 1 week, the patient's edema disappeared. His weight decreased by 12 kg (Fig. 1A). The pulmonary edema showed significant improvement (Fig. 2C), and the serum albumin increased from 26.7 to 35.1 g/L (Fig. 1B), but the urinary protein count remained at 1.3–1.7 g/24 h. After the patient's general situation was obviously better, the renal biopsy was performed. The pathology showed glomeruli ischemic atrophy and intimal hyperplasia (“onion skin” like change) with significant narrow of lumen, which were compliance with malignant hypertensive glomerulosclerosis. Renal injury of MHT was clearly diagnosed. When the creatinine level was stable to about 300 μmol/L, renin-angiotensin system inhibitor (RASI) was added. The mean daytime BP was maintained at 135/85 mm Hg and the night time average BP was 120/70 mm Hg.

Four weeks later, on follow-up visit, he had normal BP readings (135/72 mm Hg), no edema and dyspnea, and normal chest X-ray (Fig. 2E). Other tests showed that serum albumin remained stable at 35.1 to 40 g/L, creatinine was about 300 μmol/L (Fig. 1), and the urinary protein was also 1.3 to 1.5 g/24 h.

## 3 Discussion

According to the Keith et al classification,^[3,4]^ MHT is a hypertensive emergency defined by the presence of severe hypertension (diastolic BP ≥ 130 mm Hg) in combination with ischemic retinal changes as grade III or IV hypertensive retinopathy (retinal hemorrhages and/or exudates, with or without papilledema). MHT is considered to be one of the most malicious forms of hypertension and has been strongly associated with poor prognosis.^[5]^ Multiple target organs may be involved, such as eye, kidney, heart, and brain.^[6,7]^ The main manifestations of kidney damage by MHT are proteinuria with/without microhematuria, and acute renal insufficiency.^[1]^ The pathologic changes of kidney include severe ischemic lesion of glomerulus, marked intimal hyperplasia (“onion skin” like change) even to lumen occlusion.^[5]^ The patient here was found to be severely hypertensive (BP 200/145 mm Hg). Fundus examination showed grade III hypertensive retinopathy, and the result of pathology showed glomeruli ischemic atrophy and intimal hyperplasia (“onion skin” like change) with significant narrow of lumen. Combing the level of BP, fundus examination and renal pathology, MHT is definitely diagnosed.

The CLS was first proposed by Clarkson in 1960,^[8]^ with manifestations of hypoalbuminemia, diffused pitting edema, exudative serous cavity effusions, noncardiogenic pulmonary edema, as well as hypovolemic hypotension, and even shock with multiple-organ failure in some cases.^[9]^ There are a lot of diseases that can lead to CLS. The most common serious disease is sepsis. Except sepsis, other diseases can also result in CLS, such as engraftment syndrome, viral hemorrhagic fevers, and snakebite envenomation.^[2]^ CLS caused by various diseases shares the same underlying pathophysiologic abnormality, damage of capillary endothelial cells and increase in capillary permeability to albumin.^[2,10]^ In a study of CLS, Atkinson et al estimated that the capillaries are unable to retain macro-molecules smaller than 200 kDa (the molecular weight of albumin is 66.5 kDa). In their study, Atkinson et al measured about 30% to 50% loss of albumin from the intravascular space in CLS.^[11,12]^ As a result, there is a loss of protein-rich fluid from the intravascular to the interstitial space, which leads to intravascular volume depletion, resulting in decrease of effective circulating blood volume. Besides, the loss of intravascular protein-rich fluid can also lead to the decreasing of plasma colloid osmotic pressure, and water move from intravascular to the interstitial space, resulting in tissue edema, even exudative serous cavity effusions.^[2,10]^

There are some other causes of edema, such as nephrotic syndrome, liver dysfunction, and cardiac insufficiency. Although the patient had hypoalbuminemia, he had only mild proteinuria <2 g/d, which was not conform to the nephrotic syndrome. He had no previous history of liver disease, and the test of liver function was normal. On admission, he had dyspnea, chest distress and breath holding, and was in semi decubitus position. After diuretic therapy, the symptoms of dyspnea and chest distress were improved quickly. At first glance, all of above clinical manifestations were consistent with congestive heart failure. However, the TTE indicated a completely normal heart function and serial electrocardiograms and cardiac troponins excluded myocardial infarction. In addition, what strange is that, in the first, with the improvement of symptom, there was no change of pulmonary edema signs on imaging scan, and the reasons that cause hypoalbuminemia and pulmonary edema have to be found. Taking into consideration of his severe edema, decreased serum albumin, nonmassive proteinuria and normal LVEF, we speculated that the diagnosis maybe CLS.

At present, there is no case report of CLS caused by MHT. We have known that the function of endothelium plays a pivotal role in the adjusting of vascular biology. The endothelium is the primary site of injury in MTH.^[13]^ Therefore, we think that MHT can also result in CLS. As reported before,^[14]^ high-dose IVIg (1 g/kg/d) has been used with CLS. IVIgs have several immunomodulatory actions: an anti-idiotype effect against autoantibodies and induction of Fc-mediated blockade of Fc receptors on leukocytes. IVIg can also prevent complement from mediating tissue damage. Moreover, IVIg react with a number of molecules expressed on T-cell, B-cell, and monocyte membranes, and are implicated in the control of autoreactivity and tolerance induction. Considering the mechanisms probably involved in CLS, the pathogenic mechanism has not yet been clearly established. In this case, we had given only 5 g IVIg (100 mg/kg/d) to this patient for attempt, but the function of IVIg here is not clear. In addition, we also used the drugs of sulodexide and RASI to repairing vascular endothelium and improving the function of vascular endothelium. Before discharge, the patient's edema disappeared, the chest X-ray showed obviously improvement, and the level of serum albumin increased to 35.1 g/L simultaneously.

In the future, we should pay more attention to CLS induced by MHT, try to diagnose it as soon as possible, and give prompt treatment to CLS and primary disease.

## Author contributions

**Data curation:** Guoqin Wang.

**Formal analysis:** Lijun Sun, Hongrui Dong.

**Project administration:** Yipu Chen.

**Writing – original draft:** Xuejiao Liu.

**Writing – review & editing:** Hong Cheng.
